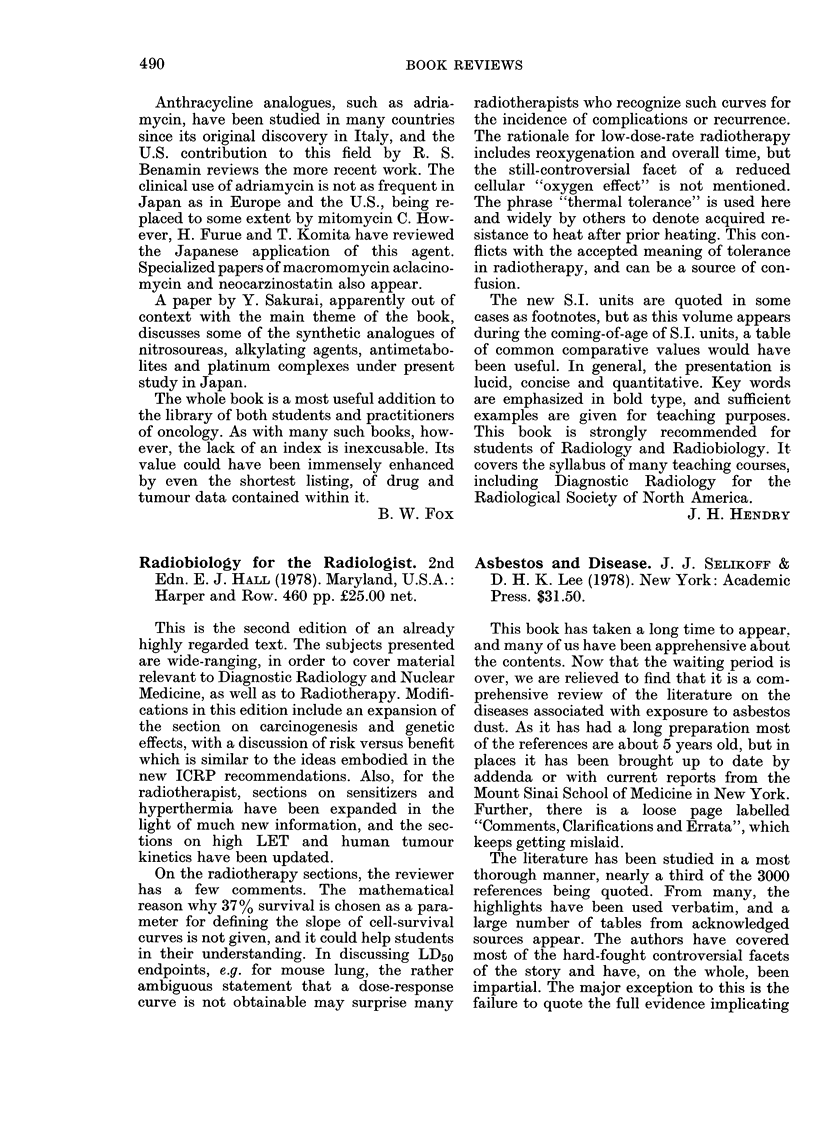# Radiobiology for the Radiologist

**Published:** 1979-04

**Authors:** J. H. Hendry


**Radiobiology for the Radiologist.** 2nd Edn. E. J. Hall (1978). Maryland, U.S.A.: Harper and Row. 460 pp. £25.00 net.

This is the second edition of an already highly regarded text. The subjects presented are wide-ranging, in order to cover material relevant to Diagnostic Radiology and Nuclear Medicine, as well as to Radiotherapy. Modifications in this edition include an expansion of the section on carcinogenesis and genetic effects, with a discussion of risk versus benefit which is similar to the ideas embodied in the new ICRP recommendations. Also, for the radiotherapist, sections on sensitizers and hyperthermia have been expanded in the light of much new information, and the sections on high LET and human tumour kinetics have been updated.

On the radiotherapy sections, the reviewer has a few comments. The mathematical reason why 37% survival is chosen as a parameter for defining the slope of cell-survival curves is not given, and it could help students in their understanding. In discussing $LD_{50}$ endpoints, *e.g.* for mouse lung, the rather ambiguous statement that a dose-response curve is not obtainable may surprise many radiotherapists who recognize such curves for the incidence of complications or recurrence. The rationale for low-dose-rate radiotherapy includes reoxygenation and overall time, but the still-controversial facet of a reduced cellular "oxygen effect" is not mentioned. The phrase "thermal tolerance" is used here and widely by others to denote acquired resistance to heat after prior heating. This conflicts with the accepted meaning of tolerance in radiotherapy, and can be a source of confusion.

The new S.I. units are quoted in some cases as footnotes, but as this volume appears during the coming-of-age of S.I. units, a table of common comparative values would have been useful. In general, the presentation is lucid, concise and quantitative. Key words are emphasized in bold type, and sufficient examples are given for teaching purposes. This book is strongly recommended for students of Radiology and Radiobiology. It covers the syllabus of many teaching courses, including Diagnostic Radiology for the Radiological Society of North America.

J. H. Hendry